# A Multi-Component Day-Camp Weight-Loss Program Is Effective in Reducing BMI in Children after One Year: A Randomized Controlled Trial

**DOI:** 10.1371/journal.pone.0157182

**Published:** 2016-06-30

**Authors:** Kristian Traberg Larsen, Tao Huang, Mathias Ried-Larsen, Lars Bo Andersen, Malene Heidemann, Niels Christian Møller

**Affiliations:** 1 Centre of Research in Childhood Health (RICH), Department of Sports Science and Clinical Biomechanics, University of Southern Denmark, Odense, Denmark; 2 The Centre of Inflammation and Metabolism (CIM) and The Centre for Physical Activity Research (CFAS), The Danish Diabetes Academy, Rigshospitalet, Copenhagen, Denmark; 3 Department of Sports Medicine, Norwegian School of Sport Sciences, Oslo, Norway; 4 Department of Pediatrics, Hospital Lillebaelt, Kolding, Denmark; 5 The Danish Diabetes Academy, Odense, Denmark; Institute of Preventive Medicine, DENMARK

## Abstract

The objective of the present study was to evaluate the effectiveness of a one-year multi-component immersive day-camp weight-loss intervention for children with overweight and obesity. The study design was a parallel-group randomized controlled trial. One hundred fifteen 11-13-year-old children with overweight and obesity were randomized into either: A six-week day-camp intervention arm focusing on increased physical activity, and healthy diet followed by a subsequent one-year family-based intervention, or a standard intervention arm consisting of one weekly exercise session for six weeks. Body mass index (BMI) was the primary outcome. BMI z-score, clustered cardiovascular risk z-score, and body composition were secondary outcomes. All outcomes were measured at baseline, six week-, and 52 week follow-up. After six weeks, children from the day-camp intervention arm had improved their BMI (-2.2 kg/m^2^ (95% CI -2.6 to -1.7, P<0.001)) and all secondary outcomes when compared to the children from the standard intervention arm. After 52 weeks, the day-camp intervention arm had a lower BMI (-1.2 kg/m^2^ (95% CI -1.8 to -0.5, P = 0.001)), and BMI z-score (-0.20 (95% CI -0.35 to -0.05, P = 0.008)), and clustered cardiovascular risk z-score (-0.23 (95% CI -0.37 to -0.08, P = 0.002)) compared to the standard intervention arm. No group differences were detected in body composition after 52 weeks. This study shows that the day-camp intervention arm is effective in reducing BMI and improving the metabolic health of children with overweight and obesity. However, the effects seem to be diminishing over time.

## Introduction

Only a few well-designed randomized clinical trials have been conducted on the reversal of overweight and obesity in childhood and adolescence [1]. Consequently, no consensus have yet been reached on how to design and conduct an effective concept as the sparse number of studies vary substantially in methods and quality [1]. Until now, the most promising treatment strategies have been multi-component approaches, which include combined diet, physical activity, parental involvement, and behavioral components [1, 2].

Immersive weight-loss interventions in camp settings have the potential of providing an intensive multi-component effort in relatively controlled surroundings [3]. A number of studies have previously evaluated the effects of camp-based interventions for children and adolescents with overweight and obesity [4–11]. Despite promising results, most of the existing evaluations of camp interventions have undertaken non-randomized approaches and short-term follow-up periods, thus, making it difficult to determine causality [3]. In order to prevent future obesity, and the subsequent adverse health outcomes, there is an essential need for implementing more effective child-directed weight-loss interventions by adopting successful approaches that take place in real-world settings and to evaluate these in well-designed effectiveness trials.

The primary aim of the present study was to evaluate the effect of a camp-based intervention on children’s body mass index (BMI). In order to shed light over a broad variety of health related aspects of the intervention program BMI z-score (zBMI), clustered cardiovascular risk (CCR) z-score, and body composition were secondary outcomes. We hypothesized that a day-camp intervention was superior in reducing BMI after six weeks as well as after one year compared to a standard intervention. Secondarily, we expected the children participating in the day-camp to improve their CCR z-score compared to the standard intervention.

## Methods

### Study design

The Odense Overweight Intervention Study (OOIS) was a parallel-group comparative effectiveness trial with one-year follow-up. A study protocol with a detailed description of methods, intervention components, and analysis strategies has previously been published [12]. The study protocol was approved by The Regional Scientific Ethical Committee for Southern Denmark (Approval number: S-20120015) and at ClinicalTrial.gov (Registration number: NCT01574352). Written consent was collected from legal guardians. Due to consideration of the school curriculum, it was necessary to perform the randomization four weeks prior to the baseline measurements. The trial was conducted as open-labelled since blinding of participants was not found practically possible. Assessors were blinded during all assessments. The random allocation sequence (1:1), enrolment, and assignment of participants were done by a researcher (KTL) and the municipal staff.

### Sample size calculation

Based on a pilot day-camp conducted in 2011 by the Odense Municipality, Denmark, a minimum difference in BMI of 1.5 kg/m^2^ between the intervention arms over 12 months was expected. Achieving a power of 80%, and with an expected 30% attrition, a minimum of 49 participants in each arm should be recruited (total n = 98).

### Eligibility and recruitment

In total, 3 750 fifth grade primary school children (i.e. 91.3% of all fifth graders) in the Municipality of Odense had their body height, -weight, and waist circumference assessed by school nurses as a part of the mandatory health examination. Of these, 541 (14.4%) children were overweight and 92 (2.5%) were obese. Children with a BMI above the limit for overweight according to the International Obesity Task Force (IOTF) [13], were offered to participate in the OOIS. The recruitment took place during two school years (2011–12 and 2012–13). Exclusion criteria included:

1. Children participated in other intervention research involving cardiovascular risk management.
2. Children that did not attend a regular school due to personal problems of psycho-social nature.
3. Children taking any medications, during three months prior to entering the study, which are known to affect weight status.
4. Children with a known endogenous cause of overweight.
5. Children with a motor-control handicap that prohibited normal participation in physical activity.

### Day-camp intervention arm (DCIA)

The day camp was located in the city of Odense, Denmark, and took place from mid-May to the end of June in 2012 and 2013, respectively. The camp lasted for six consecutive weeks, seven days a week. The children arrived every morning at 7 a.m. and left at 8.30 p.m. Except for commuting between home and the day-camp, children typically stayed at home with their families outside this time period. Each day the children were engaged in minimum three hours of exercise with a focus on physical activity enjoyment and motivation (e.g. dancing, team building, and alternative ball-games), one hour of health classes (focused on theory and behavior change), and one hour of homework assignment (as the intervention took place during school weeks). Trained instructors planned and conducted the intervention components and supported the children during the day-camp. Meals were prepared, supervised and guided by the camp instructors according to the national Danish dietary recommendations [14], but no caloric restriction was enforced. During the intervention period, parents received written information about the intervention, healthy cooking in the household, and advice on how best to support the child’s health behavior. Furthermore, parents participated in a dietary course, with the children at the camp, led by a certified dietician.

#### Subsequent family-based intervention

After the six week intervention, a family-based intervention including four joint meetings during the subsequent 46 weeks was conducted. The meetings were led by trained school nurses and instructors from the day-camp intervention. At all meetings, the families discussed and shared experiences related to a chosen topic. After the second meeting, an “activity day” was arranged for the children by the camp instructors in order to support and motivate the children for the remaining intervention period.

#### Theoretical approach

The theoretical framework of the intervention program was based on a psychosocial model of planned behavior change and the trans-theoretical model at the individual level through systematic use of group processes [15].

### Standard intervention arm (SIA)

For comparison with the day-camp program, a short term and low intensive weight-loss program was constructed. The standard intervention consisted of one weekly exercise session (two hours duration) for six weeks, as well as a single health and lifestyle educational session for the parents, delivered by a dietician and a physical activity specialist. The standard intervention program was taking place simultaneously with the day-camp program and ended after six weeks.

### Study outcomes

A description of the measurements and appurtenant time points are presented in Table 1. BMI and secondary outcomes were measured on the Odense University Hospital and on the University of Southern Denmark in Odense at: 1) baseline, 2) six week follow-up, and 3) 52 week follow-up. Background, lifestyle, and child behavior questionnaires (in paper form) for the parents were collected at baseline and 52 weeks follow-up. BMI has earlier proven to be a reasonable assessment of change of adiposity in growing children [16]. The potential sex, age and growth related confounders associated with BMI are levelled out when using a randomized design. Secondary outcomes were zBMI, body composition by total body-fat percentage and abdominal fat percentage, and CCR z-score. zBMI was calculated based on the IOTF growth charts [17]. The CCR z-score was modified based on the definition by Andersen et al. [18]. Included risk factors were systolic blood pressure, triglycerides, total cholesterol to high-density lipoprotein (HDL) ratio, insulin resistance by homeostasis model analysis—insulin resistance (HOMA-IR), abdominal fatness by waist circumference, and cardiorespiratory fitness. HOMA-IR was calculated according to the original formula by Matthews et al. [19]. Waist circumference, triglycerides, HOMA-IR, and total cholesterol to HDL ratio were slightly skewed and, consequently, log transformed. Cardiorespiratory fitness scores were reversed in order to fit the direction of the CCR z-score. Subsequently, z-scores of the individual risk factors were summed to construct the CCR z-score. A low CCR z-score was considered healthier than a high score. The outcomes were also examined post hoc according to dichotomized BMI (children with overweight/obesity versus without overweight/obesity) using the IOTF cut points [13]. Children’s physical activity was assessed with Actigraph GT3X+ accelerometers during the day-camp for seven consecutive days. Food intake during the day-camp was assessed objectively by weight and photographical registration.

**Table 1:** Description of study constructs, the applied measurement methods, and the time points for the measurements.

Construct	Method of measurement	Measurement time points			
Primary outcome		*Baseline*	*During camp*	*6 week follow up*	*52 week follow up*
BMI	Height assessed without footwear and weight in underwear on a Soehnle 7700 Professional Medical electronic scale (Murrhardt, Germany).	√		√	√
**Secondary outcomes and explanatory variables**					
Anthropometrics and body composition	The waist circumference was assessed between the lower costal margin and the iliac crest to the nearest 0.5 cm, at the end of a gentle expiration. Dual energy X-ray absorptiometry (DXA) was performed by an experienced operator on a GE Lunar Prodigy (GE Medical Systems, Madison, WI), equipped with ENCORE software (version 12.3, Prodigy; Lunar Corp, Madison, WI).	√		√	√
Pubertal development	Pubertal development was assessed according to Tanner by self-evaluation to avoid violating the child’s intimacy. During self-evaluation the child privately estimated genitalia and breast development based on drawings (provided in S1 Appendix and S2 Appendix).	√		√	√
Cardiorespiratory fitness	By a progressive bicycle ergometer protocol (Monark Ergomedic 839e) until total exhaustion with indirect calorimetry (Innovision, AMIS 2001) and a Polar, RS800CX heart rate monitor.	√		√	√
Blood pressure	Blood pressure was assessed after 5 min of rest in a sitting position on the left upper arm with an automatic blood pressure monitor (Welch Allyn 300 series).	√		√	√
Blood measurements	After overnight fast, blood samples were drawn in the morning from the antecubital vein (right arm).The participants were lying supine during blood collection. Samples for serum were left in room temperature for 30 min. Samples for plasma (EDTA) were centrifuged as soon as possible, and they were placed in an icebox (within one hour) before centrifugation. All samples were centrifuged at 2500G for 10 min. Then the samples were stored at -80°C until analysis.Insulin was analyzed with AlphaLISA® immunoassay kits (PerkinElmer, Waltham, MA, USA) in 384 microplate platform. The serum glucose and lipids were measured with Architect C16000 clinical chemistry analyzer (Abbott Diagnostics, Illinois, USA). Glucose was analyzed using Hexokinase/G-6-PDH methods. HDL-C and LDL-C were measured using homogenous enzymatic colorimetric analysis. TC and TG were analyzed using enzymatic colorimetric analysis.	√		√	√
Parent’s lifestyle, ethnicity, and socio-economic status	By letter distributed questionnaire. Parents socio-economic status was based on the mothers highest educational level.	√			√
Physical activity	Assessed by hip-worn accelerometer Actigraph GT3X+ for ten consecutive days for habitual physical activity, for seven consecutive days for estimated activity level during the day camp (SCIA only), and for two sessions of exercise sessions for the SIA.	√	√		√
Food intake during camp	Assessed by food registration for two entire days. For every meal the child had their food weighed and photographed for subsequent analyses of energy and nutrition content, with Winfood 4.0 software, by trained and qualified personal.		√		

### Data analyses and statistics

The Shapiro–Wilk test was used to test for normality. Frequencies, means with standard deviations, or medians with inter-quartile ranges were calculated for the demographics and anthropometric measures depending on normality of the data. T-tests for normal distributed data, Wilcoxon rank-sum tests for non-normal distributed data, and Chi^2^ tests for categorical data were used to determine if any significant differences between intervention arms- or year groups were present (Table 2). For retrieving the study outcomes the groups were analyzed as they were randomized. However, the randomization took place before baseline measurements (cf. “Study design” paragraph) due to practical circumstances. As nine children dropped out after the randomization and before the baseline measurements, they could not be included in the analyses. Hence, a true intention-to-treat analysis, as planned, was not possible to perform. In all other aspects, the analysis strategy follows the study protocol [12].

**Table 2:** Baseline characteristics.

	Total	Day Camp Intervention Arm	Standard Intervention Arm	P-values
**Total N (male %)**	106 (44.3)	55 (47.3)	51 (41.2)	^C2^ 0.528
**Age (years)**	(n = 106)	(n = 55)	(n = 51)	
*Mean (SD)*	12.0 (0.4)	12.0 (0.4)	12.0 (0.4)	^TT^ 0.344
**Ethnic Danish (%)**	66.0‡	70.6	61.8	^C2^ 0.465
**Socio-economic status ♂ / ♀ (N)†**	(n = 99)	(n = 52)	(n = 47)	
*1*	11 / 14	7 / 8	4 / 6	
*2*	18 / 21	11 / 13	7 / 8	
*3*	12 / 23	6 / 7	6 / 16	^C2^ 0.076
**Pubertal stage ♂ / ♀ (N)**	(n = 106)	(n = 55)	(n = 51)	
*1*	4 / 0	3 / 0	1 / 0	
*2*	24 / 4	13 / 2	11 / 2	
*3*	17 / 37	9 / 18	8 / 19	
*4*	2 / 15	1 / 9	1 / 6	
*5*	0 / 3	0 / 0	0 / 3	^C2^ 0.339
**Body height (cm)**	(n = 106)	(n = 55)	(n = 51)	
*Mean (SD)*	156.0 (6.1)	156.4 (6.6)	155.5 (5.7)	^TT^ 0.465
**Body weight (kg)**	(n = 106)	(n = 55)	(n = 51)	
*Median (IQR)*	60.1 (53.9–65.5)	61.3 (55.4–66.2)	59.2 (52.4–62.9)	^WR^ 0.117
**BMI (kg/m**^**2**^**)**	(n = 106)	(n = 55)	(n = 51)	
*Median (IQR)*	24.3 (22.6–26.9)	24.8 (22.8–27.1)	23.9 (22.5–26.9)	^WR^ 0.215
**BMI z-score**	(n = 106)	(n = 55)	(n = 51)	
Mean (SD)	1.94 (0.49)	1.99 (0.46)	1.87 (0.51)	^TT^ 0.209
**CCR z-score**	(n = 106)	(n = 55)	(n = 51)	
*Mean (SD)*	0.00 (0.62)	0.12 (0.62)	-0.13 (0.60)	^TT^ 0.044*
**Waist circumference (cm)**	(n = 106)	(n = 55)	(n = 51)	
*Median (IQR)*	83.0 (78.5–88.0)	84.5 (80.5–89.0)	81.5 (77.0–85.0)	^WR^ 0.015*
**Total body fat (%)**	(n = 104)	(n = 55)	(n = 49)	
*Mean (SD)*	39.3 (6.2)	39.5 (6.2)	39.2 (6.2)	^TT^ 0.810
**Abdominal fat (%)**	(n = 104)	(n = 55)	(n = 49)	
*Median (IQR)*	47.8 (43.8–52.8)	47.9 (44.5–53.8)	47.0 (42.6–52.5)	^WR^ 0.587
**Fat free mass (kg)**	(n = 104)	(n = 55)	(n = 49)	
*Median (IQR)*	34.0 (30.6–36.3)	34.8 (31.0–37.3)	33.3 (30.0–35.2)	^WR^ 0.055
**Fitness (ml O2/min/kg)**	(n = 86)	(n = 80)	(n = 75)	
*Mean (SD)*	34.0 (5.3)	33.2 (5.4)	34.8 (5.1)	^TT^ 0.167
**Systolic blood pressure (mm Hg)**	(n = 104)	(n = 55)	(n = 49)	
*Mean (SD)*	105.4 (8.1)	106.6 (7.6)	103.9 (8.5)	^TT^ 0.088
**Overweight category**	(n = 106)	(n = 55)	(n = 51)	
*Normal weight (N)*	9	3	6	
*Overweight (N)*	67	36	31	
*Obese (N)*	30	16	14	^C2^ 0.507
**HDL/Total Cholesterol ratio**	(n = 99)	(n = 54)	(n = 45)	
*Median (IQR)*	3.27 (2.91–3.78)	3.29 (2.95–3.90)	3.18 (2.87–3.54)	^WR^ 0.307
**Triglycerides (mmol/L)**	(n = 99)	(n = 54)	(n = 45)	
*Median (IQR)*	0.80 (0.59–1.21)	0.80 (0.66–1.21)	0.83 (0.57–1.14)	^WR^ 0.516
**Glucose (mmol/L)**	(n = 93)	(n = 52)	(n = 41)	
*Median (IQR)*	5.03 (4.79–5.26)	5.09 (4.86–5.33)	4.97 (4.71–5.12)	^WR^ 0.048*
**Insulin (μIU/mL)**	(n = 92)	(n = 52)	(n = 40)	
*Median (IQR)*	8.90 (6.39–12.50)	9.35 (6.56–12.33)	8.19 (6.33–13.41)	^WR^ 0.654
**Total cholesterol (mmol/L)**	(n = 99)	(n = 54)	(n = 45)	
*Median (IQR)*	4.23 (3.81–4.71)	4.38 (3.90–4.81)	3.93 (3.70–4.48)	^WR^ 0.030*
**HDL cholesterol (mmol/L)**	(n = 99)	(n = 54)	(n = 45)	
*Median (IQR)*	1.30 (1.14–1.48)	1.32 (1.14–1.50)	1.27 (1.11–1.47)	^WR^ 0.576
**HOMA-IR score**	(n = 92)	(n = 52)	(n = 40)	
*Median (IQR)*	2.00 (1.40–2.89)	2.06 (1.46–2.89)	1.66 (1.32–2.86)	^WR^ 0.498

Means with standard deviations (SD) for normal distributed data and medians with inter quartile ranges (IQR) for non-normal distributed data are presented for each intervention arm and for the total sample. To test for group differences at baseline T-tests for normal distributed data, Wilcoxon rank-sum tests for non-normal distributed data, and Chi^2^ tests for categorical data were applied.

^TT^ = T test.

^C2^ = Chi^2^ test.

^WR^ = Wilcoxon rank-sum test.

* = P<0.05.

† = Based on the mothers’ education level.

‡ = There is significant difference between year groups.

BMI = body mass index. CCR = Clustered cardiovascular risk. HDL = high-density lipoprotein. HOMA-IR = homeostasis model analysis—insulin resistance.

We used linear mixed-effects modelling for repeated measures to estimate the difference in the mean change of the characteristics between intervention arms. This method has shown to be preferable in longitudinal analyses and can be performed without any imputations of missing data [20]. Furthermore, estimates from the linear mixed model were extracted to report within-group changes. Maximum likelihood estimation was used for all models [21]. Akaike information criterion and Bayesian information criterion determined whether random intercept or random slope models were preferred. The results (difference in changes) from log-transformed outcomes were exponentiated as ratios of geometric means of changes between the two groups. For sensitivity analyses, compliance was determined according to predefined decision rules as described in the study protocol [12]. Significance level was set at P<0.05. For all statistical analysis, Stata version 12.1 SE (StataCorp LP, College Station, TX, USA) was used.

## Results

### Baseline characteristics and trial flow

**Fig 1:**
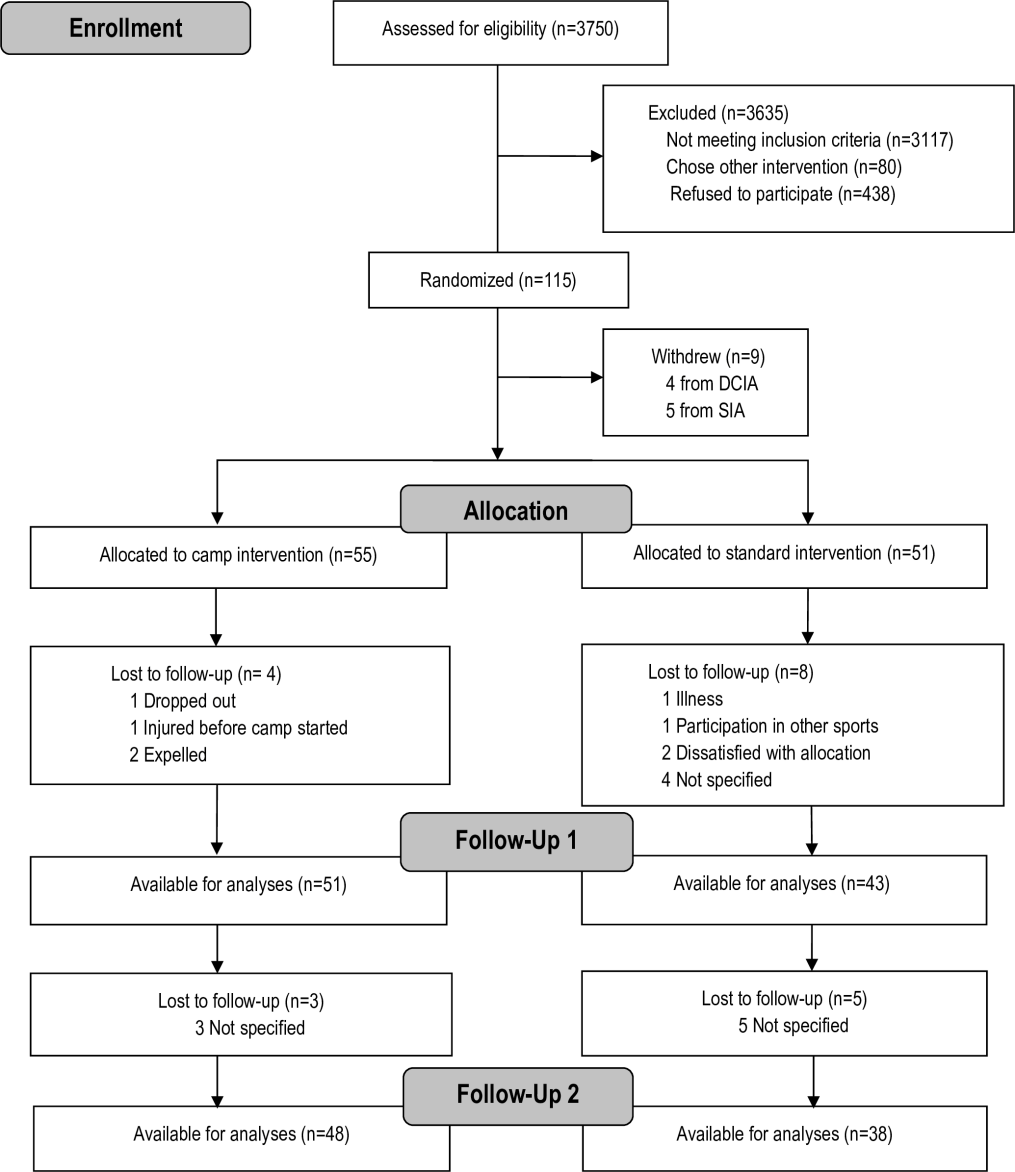
Flow of participants.

Baseline characteristics of the participants are shown in Table 2. Group differences at baseline were present with respect to waist circumference (3.0 cm larger in DCIA, P = 0.015), CCR z-score (0.25 larger in DCIA, P = 0.048), fasting blood glucose (0.12 mmol/L larger in DCIA, P = 0.048), and fasting blood total cholesterol (0.45 mmol/L larger in DCIA, P = 0.030). No other differences were found between children attending the two intervention arms at baseline. Fig 1 shows the flow of participants during the trial. The loss to follow-up was 32% and 19% in the SIA and DCIA, respectively. In the SIA, children lost to follow-up had a higher waist circumference at baseline (P = 0.034). Children from the DCIA had a higher prevalence of non-Danish ethnicity (P = 0.038). In other aspects children lost to follow-up were comparable to other children.

### Outcomes

#### Effects on BMI, zBMI, and body composition

Table 3 presents the results from the primary analyses on BMI, zBMI, percentage body fat and percentage abdominal fat. The relative difference in in change for BMI between the groups after 52 weeks was -1.2 kg/m^2^ (95% CI -1.8 to -0.5, P = 0.001) in favor of the DCIA. This pattern was similar for zBMI (-0.20 (95% -0.35 to -0.05, P = 0.008)). A significant difference in change between intervention arms in body fat percentage and abdominal body fat percentage could be observed at six weeks in favor of the DCIA, but not at 52 weeks. No differences between intervention arms were observed in the change of fat free mass (FFM) at either follow-up assessment. A significant within-group decrease in zBMI was observed in the SIA at both six and 52 week follow-up.

**Table 3:** The intervention effects on BMI, BMI z-score, and body composition.

	Mean (SD), N = SIA/DCIA			Within group change at 6 weeks	Within group change at 52 weeks	Difference in change at 6 weeks		Difference in change at 52 weeks	
Variable	Baseline	6 weeks	52 weeks	Mean(95% CI)	Mean(95% CI)	Mean(95% CI)	P-value	Mean(95% CI)	P-value
**BMI (kg/m2)**	N = 51/55	N = 43/51	N = 38/48						
**Standard**	24.5 (2.9)	24.2 (3.1)	24.8 (3.7)	-0.2 (-0.5 to 0.1)	0.1 (-0.4 to 0.6)	-2.2 (-2.6 to -1.7)	<0.001	-1.2 (-1.8 to -0.5)	0.001
**Day Camp**	25.2 (2.8)	22.7 (2.6)	23.8 (3.1)	-2.4 (-2.7 to -2.1)*	-1.1 (-1.5 to -0.6)*				
**BMI z-score**	N = 51/55	N = 43/51	N = 38/48						
**Standard**	1.87 (0.51)	1.77 (0.56)	1.73 (0.66)	-0.08 (-0.15 to -0.01)*	-0.19 (-0.30 to -0.08)*	-0.44 (-0.54 to -0.34)	<0.001	-0.20 (-0.35 to -0.05)	0.008
**Day Camp**	1.99 (0.46)	1.45 (0.58)	1.53 (0.63)	-0.52 (-0.59 to -0.45)*	-0.39 (-0.49 to -0.29)*				
**Total body fat (%)**	N = 49/55	N = 44/50	N = 39/47						
**Standard**	39.2 (6.2)	38.1 (7.0)	37.3 (8.1)	-1.3 (-2.3 to -0.4)*	-2.0 (-3.4 to -0.7)*	-5.1 (-6.4 to -3.8)	<0.001	-1.8 (-3.7 to 0.1)	0.062
**Day Camp**	39.5 (6.2)	32.9 (7.4)	34.4 (7.3)	-6.5 (-7.3 to -5.6)*	-3.8 (-5.1 to -2.6)*				
**Abdominal fat (%)**	N = 49/55	N = 44/50	N = 39/47						
**Standard**	47.5 (7.3)	45.6 (8.0)	44.2 (9.9)	-1.8 (-3.2 to -0.5)*	-3.0 (-4.8 to -1.1)*	-7.2 (-9.0 to -5.3)	<0.001	-2.1 (-4.6 to 0.4)	0.093
**Day Camp**	48.0 (7.1)	38.9 (9.8)	41.5 (9.6)	-9.0 (-10.2 to -7.8)*	-5.1 (-6.8 to -3.4)*				
**Fat free mass (kg)**	N = 49/55	N = 44/50	N = 39/47						
**Standard**	33.2 (4.2)	34.3 (3.9)	37.3 (4.7)	1.0 (0.4 to 1.6)*	3.7 (3.1 to 4.4)*	-0.5 (-1.3 to 0.8)	0.276	-0.5 (-1.3 to 0.8)	0.289
**Day Camp**	34.7 (4.1)	35.2 (4.4)	38.1 (5.1)	0.6 (-0.0 to 1.1)	3.2 (2.7 to 3.8)*				

BMI, BMI z-score, percentage body fat and percentage abdominal fat at baseline and difference in group changes after 6 and 52 weeks using two-sided linear mixed effects models for repeated measures.

* = P<0.05.

#### Effects on clustered cardiovascular risk

As presented in Table 4, differences in CCR z-score between intervention arms were present after both six and 52 weeks. The CCR z-score had increased within the SIA both at both follow up assessments compared to the baseline value. In the DCIA, a within group decrease was present after six weeks, but not after 52 weeks. The underlying components for the CCR z-score are depicted in Table 4. Systolic blood pressure and HDL/total cholesterol ratio improved in the DCIA compared to the SIA at both follow-up measurements. Cardiorespiratory fitness and waist circumference improved in the DCIA compared to the SIA after six weeks but not after 52 weeks, and no group differences were observed at either follow-up measurement in triglycerides and HOMA-IR score.

**Table 4:** The intervention effects on clustered cardiovascular risk and the contributing components.

	Mean (SD), N (SIA/DCIA)			Within group change at 6 weeks	Within group change at 52 weeks	Difference in change at 6 weeks		Difference in change at 52 weeks	
Variable	Baseline	6 weeks	52 weeks	Mean(95% CI)	Mean(95% CI)	Mean(95% CI)	P-value	Mean(95% CI)	P-value
**CCR z-score**	N = 51/55	N = 50/55	N = 49/55						
**Standard**	-0.13 (0.60)	0.14 (0.71)	0.04 (0.70)	0.28 (0.17 to 0.38)*	0.19 (0.08 to 0.29)*	-0.41 (-0.55 to -0.26)	<0.001	-0.23 (-0.37 to -0.08)	0.002
**Day Camp**	0.12 (0.62)	-0.01 (0.57)	0.08 (0.59)	-0.13 (-0.23 to 0.03)*	-0.04 (-0.14 to 0.06)				
**Fitness (ml O2/min/kg)**	N = 41/45	N = 35/45	N = 32/43						
**Standard**	34.8 (5.1)	35.9 (6.0)	35.4 (5.5)	1.0 (-0.4 to 2.4)	0.2 (-1.3 to 1.7)	3.1 (1.2 to 5.0)	0.001	1.0 (-0.9 to 2.9)	0.301
**Day Camp**	33.2 (5.4)	38.1 (6.9)	35.4 (6.0)	4.1 (2.8 to 5.3)*	1.2 (-0.0 to 2.5)				
**Systolic blood pressure (mm Hg)**	N = 49/55	N = 44/51	N = 38/47						
**Standard**	103.9 (8.5)	103.1 (7.7)	105.0 (8.6)	-1.0 (-2.9 to 0.9)	0.8 (-1.2 to 2.8)	-5.4 (-7.9 to -2.8)	<0.001	-3.1 (-5.7 to -0.4)	0.026
**Day Camp**	106.6 (7.6)	100.1 (6.9)	103.9 (6.3)	-6.3 (-8.1 to -4.6)*	-2.2 (-4.0 to -0.4)*				
**Waist circum-ference (cm)**	N = 51/55	N = 43/51	N = 38/48						
**Standard**	82.4 (8.1)	82.0 (8.8)	79.8 (9.0)	-0.3 (-1.8 to 1.1)	-2.7 (-4.6 to -0.8)*	-5.4 (-7.4 to -3.5)	<0.001	-2.0 (-4.5 to 0.6)	0.132
**Day Camp**	85.5 (7.8)	79.4 (8.6)	79.8 (8.3)	-5.8 (-7.1 to -4.4)*	-4.7 (-6.4 to -3.0)*				
**Triglycerides (mmol/L)†**	N = 45/54	N = 36/47	N = 32/42						
**Standard**	0.90 (0.44)	1.01 (0.70)	0.87 (0.52)	1.10 (0.93 to 1.28)	0.98 (0.83 to 1.15)	0.85 (0.69 to 1.05)	0.126	1.02 (0.82 to 1.26)	0.886
**Day Camp**	1.03 (0.60)	0.89 (0.40)	1.00 (0.50)	0.93 (0.81 to 1.07)	0.99 (0.86 to 1.15)				
**Total/HDL cholesterol ratio**	N = 45/54	N = 36/47	N = 32/42						
**Standard**	3.3 (0.7)	3.4 (0.8)	3.4 (1.1)	0.07 (-0.10 to 0.23)	0.10 (-0.07 to 0.27)	-0.30 (-0.52 to -0.07)	0.009	-0.58 (-0.81 to -0.35)	<0.001
**Day Camp**	3.5 (0.8)	3.2 (0.6)	3.0 (0.7)	-0.23 (-0.38 to -0.08)*	-0.48 (-0.63 to -0.32)*				
**Glucose tolerance (HOMA-IR)†**	N = 45/54	N = 36/47	N = 32/42						
**Standard**	2.7 (2.5)	2.5 (1.8)	2.4 (1.6)	0.96 (0.77 to 1.19)	0.93 (0.75 to 1.16)	0.75 (0.57 to 1.00)	0.054	1.01 (0.75 to 1.32)	0.969
**Day Camp**	2.4 (1.4)	1.7 (0.8)	2.0 (0.7)	0.72 (0.60 to 0.87)*	0.93 (0.77 to 1.12)				

Clustered cardiovascular risk z-score (CCR z-score) and the contributing components at baseline and difference in group changes after 6 and 52 weeks using linear mixed effects models for repeated measures.

* = P-value <0.05.

† = results presented in ratios after log transformation and back transformation (due to non-normal distributed data).

#### Effects on obesity status

Nine children were normal weight at baseline (three in the DCIA and six in the SIA). Including these, 23.6% of all DCIA participants had decreased from with-overweight or -obesity to normal weight after 52 weeks, compared to 7.8% of the SIA children. In the DCIA, the odds ratio for moving minimum one weight category downwards (from with-obesity to with-overweight/normal weight or from with-overweight to normal weight) after 52 weeks was 3.4 (95% CI 1.4 to 8.2, P = 0.008), and the odds ratio for staying in the same category was 0.3 (95% CI 0.1 to 0.7, P = 0.008). Sensitivity analyses excluding the nine normal-weight children showed similar odds ratios. Furthermore, per protocol analyses revealed that attendance during the day-camp and the subsequent support meetings did not affect the results.

#### Intervention compliance

During the six-week camp period, 50 out of 52 children who initiated the camp program completed the six weeks according to the predetermined acceptable attendance rate (≥85% of the total time). One child was expelled from the camp due to destructive behavior after four days, and another child dropped out (gave up) after ten days at the camp. Twenty-five children (48.1%) lived up to the predefined attendance rate during the subsequent family-based intervention period (≥4 of 6 meetings). The day-camp children had a mean energy intake of 5920 kJ/day (SD 1071 kJ/day), with no significant differences across sex, and spent approximately 90 min in moderate to vigorous physical activity per day using the cut-points from Evenson et al. [22]. One girl from the SIA reported an eating disorder at the one-year follow-up. In the SIA participants, 68% lived up to the predetermined acceptable attendance rate (minimum 4/6 sessions).

## Discussion

The main finding in the present study was that children with overweight or obesity participating in an immersive weight-loss day-camp setting significantly reduced their BMI, zBMI, and CCR z-score both after six and after 52 weeks, when compared to children participating in a low-intensity intervention. Furthermore, significant difference in changes between groups in waist circumference, cardiorespiratory fitness, total body fat, and abdominal fat were present after six weeks in favor of the DCIA, but not after 52 weeks.

### Comparison with earlier studies

Compared to similar camp-based weight-loss interventions, the BMI effect sizes observed in the present study at both post camp and one-year follow-up were slightly smaller [5, 6]. Likely reasons for this dissimilarity could be the use of dietary restrictions, more daily supervised exercise, and no randomized comparison groups in such studies. Furthermore, it is noteworthy that the participants in the present study had a lower mean BMI (mean baseline BMI = 24.3) than participants in previous camp-based programs (typical BMI approximately>30) [5, 7–9]. Huelsing et al. found that a higher initial BMI predicted a greater reduction in BMI during a camp-based intervention program [9]. Much comparable to that, we found a similar association (P = 0.003) in the DCIA during the day-camp, but not during the subsequent family-based intervention. This association was not present in the SIA at either follow-up. These findings suggest that the six-week intense day-camp period favors more overweight children, while the period following the day-camp, independent of allocation, does not favor this subgroup.

A post hoc analysis of the DCIA between six and 52 weeks revealed a within-group increase in BMI (1.3 (95% CI 0.8 to 1.8, P<0.001)) and in zBMI (0.12 (95% CI 0.03 to 0.21, P = 0.007). With substantial differences in the intervention content and intensity between day-camp and the family-based intervention, this development was not unexpected. However, as the difference in change of BMI, BMI z-score and CCR z-score approximately were reduced by half during the period from six to 52 weeks, it makes it relevant to consider how long the effects would sustain during the period following the intervention. Previous weight-loss interventions taking place outside camp-based settings have shown effects comparable to the OOIS at one-year follow-up [23, 24]. One study also investigated the weight development over a longer time period and found a gradual regain in body fat until the effects were disappeared after 24 months when compared to a sham intervention [24]. Thus, a similar development could be expected in the OOIS, both for the weight and cardiovascular risk development. In a future perspective, it would be appropriate to investigate how the family-based intervention could be further improved in order to prolong the attained effects.

Furthermore, the abrupt change in intensity from the day-camp to the less intense family-based intervention could explain some of the group differences observed in waist circumference, cardiorespiratory fitness, total body fat, and abdominal fat after six weeks in favor of the DCIA, while none of these differences were present after 52 weeks. Another contributing factor could be the somewhat unexpected within-group improvements in the SIA for zBMI, waist circumference, body fat, abdominal fat, and FFM after 52 weeks. This indicates that at least some of the participants from the SIA changed their health-related behavior in addition to what was provided during the six-week standard intervention program. Earlier evidence have shown that most children signing up for weight-loss interventions are predetermined to lose weight [25], which could be an influencing factor in the OOIS as well. This would naturally affect both intervention arms equally. A potential regression towards the mean could also influence the observed decrease of zBMI within the SIA [26]. The effect of regression towards the mean from six to 52 weeks would be expected to be marginally larger in the SIA, as the DCIA has a lower mean zBMI after six weeks [26].

### Implications for future health

Previous observational studies have found increased childhood BMI (highest quartile compared to lowest quartile) to be associated with a 130% increased risk of premature death due to endogenous causes [27]. Correspondingly, a 1-unit increase in childhood zBMI has been observed to be associated with an increased risk of coronary heart disease in adulthood [28]. Ford and colleagues stated that a decrease of minimum 0.25 zBMI is sufficient to improve the metabolic health and a higher decrease in zBMI is associated with greater health benefits [29] (the DCIA achieved a mean decrease of 0.39 (95% CI 0.49 to 0.29, P<0.001) zBMI across 52 weeks). A decrease of 0.25 zBMI is similar to a reduction of 1 kg/m^2^ or a stable body weight observed during one year in a growing child [30]. Accordingly, the improvements in BMI and zBMI observed in the DCIA can potentially play an important role in order to prevent current and future coronary heart disease risk.

The underlying mechanisms for reductions in later coronary heart disease and premature death could relate to decreased atherosclerotic development. Earlier observations show that individuals who normalize their BMI between childhood and adulthood, will reduce their risks with respect to a number of adult subclinical markers, such as carotid intima-media thickness and concentrations of blood lipids, compared to individuals consistently being classified as normal weight or with-overweight [31–33]. A gradual increase of CCR during adolescence has earlier been established in children with overweight [34]. Delaying and/or reducing the usual increase in CCR, as observed in the DCIA, could potentially postpone the atherosclerotic process and decrease the risk of adult Type 2 diabetes mellitus [33]. Another possible health benefit relates to the observed increase in FFM indicating preservation of muscle mass in the DCIA. The benefits of preserving FFM in order to maintain achieved weight loss and improvements in metabolic health are well supported in the literature [35, 36]. Furthermore, a reduction in resting energy expenditure after weight loss is the main risk factor for later regain of weight [37, 38] and resting energy expenditure is closely related to FFM in obese children [38, 39]. As the muscles express and release cytokines and peptides exerting paracrine or endocrine effects [40], the preservation (or increase) in muscle mass during a weight-loss intervention could prove pivotal in preventing future cardio-metabolic complications [40].

### Strengths and limitations

Strengths of the study include the randomized design and the pre-published study protocol containing a detailed description of the intervention program with pre-described aims and analysis strategies [41]. Furthermore, as the intervention takes place in a real-world environment, it would be expected that the external validity of the results from this study is higher compared to results observed in highly controlled efficacy trials. The assessment battery was comprehensive and performed by trained test personnel implying a high internal validity of the study. In addition, the follow-up period was longer than reported in most similar interventions [3].

A considerable limitation of the study was that allocation of participants was necessary prior to baseline measurements. Based on data from the school examinations prior to the sampling, the withdrawals (four from the DCIA and five from the SIA) did not differ significantly with respect to BMI or waist-to-height ratios compared to their participating peers. Thus, drop-out seems to be non-differential and should only impact the widths of the confidence intervals and not the effect sizes.

A list of names and personal identification numbers of the participants were supplied to the allocating researcher (KTL) by the municipality before randomization. However, the researcher did not have previous knowledge of any of the participating children, nor did he conduct the intervention. Thus, the allocation procedure was not considered to introduce bias.

Six normal-weight children were included in the trial by school nurses during the school examinations (of which two were with-overweight at baseline assessments). The normal weight children were included due to miscommunications between the municipality and a few of the school nurses. Furthermore, between school examinations and baseline measurements approximately 1/3 of all children lost ≥ 0.5 kg/m^2^ and five children moved from the with-overweight to the normal-weight category. Consequently, nine children in total were normal weight at baseline measurements. To commit to the randomization we chose not to exclude the normal-weight children from the analyses. Post hoc sensitivity analyses excluding the normal-weight children at baseline confirmed that this did not influence results (data not shown). Assuming that all included participants were with-overweight or with-obesity, larger effect sizes could be expected.

In the SIA, children with missing data had increased baseline waist circumference compared to children with complete data. This could potentially increase the mean waist circumference during the following measurements and, consequently, contribute to the diminished group differences at 52 weeks follow-up. In the DCIA, more children with missing data had a low socio-economic status (P = 0.05) and a non-Danish ethnic background (P = 0.01) compared to children with complete data. Both ethnicity and socio-economic status have earlier been observed to relate to attendance in weight-loss interventions [42, 43].

## Conclusions

In conclusion, our findings show that an intensive day-camp intervention containing motivation-enhancing activities without diet restrictions followed by a subsequent family-based intervention is applicable and effective in reducing BMI and improving metabolic health of pre-adolescent children across one year compared to a short-term low-intensity control intervention. The health related effects seem to be slowly diminishing over time, why future focus should be on sustaining health behavior during the family-based intervention. The day camp is a feasible solution that can be applied in most organizational settings, especially in a Danish context. As no consensus has yet been reached how best to reduce overweight and obesity among children and adolescents, an immersive day-camp combined with a family focus can be one of the more effective tools in order to overcome the rising problem of childhood overweight and obesity.

## Supporting Information

S1 Appendix Tanner self-evaluation for girls. (TIF)

S2 Appendix Tanner self-evaluation for boys. (TIF)
