# Supplementary figures and images for: A Multi-Component Day-Camp Weight-Loss Program Is Effective in Reducing BMI in Children after One Year: A Randomized Controlled Trial
Source: PLoS One. 2016 Jun 30;11(6):e0157182. doi: 10.1371/journal.pone.0157182 (PMC4928840; doi:10.1371/journal.pone.0157182)

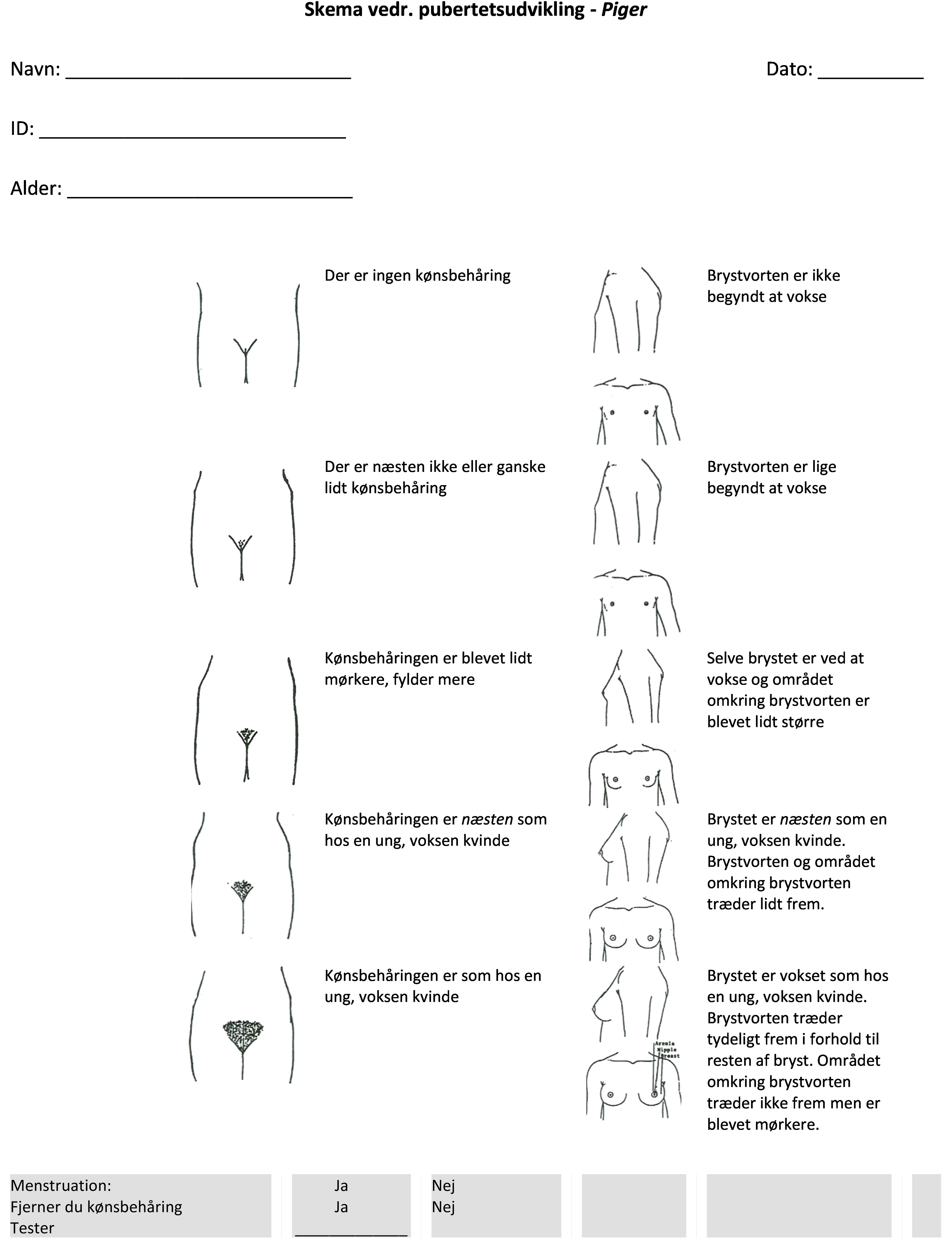

Supplement: S1 Appendix — (TIF) [file pone.0157182.s001.tif]

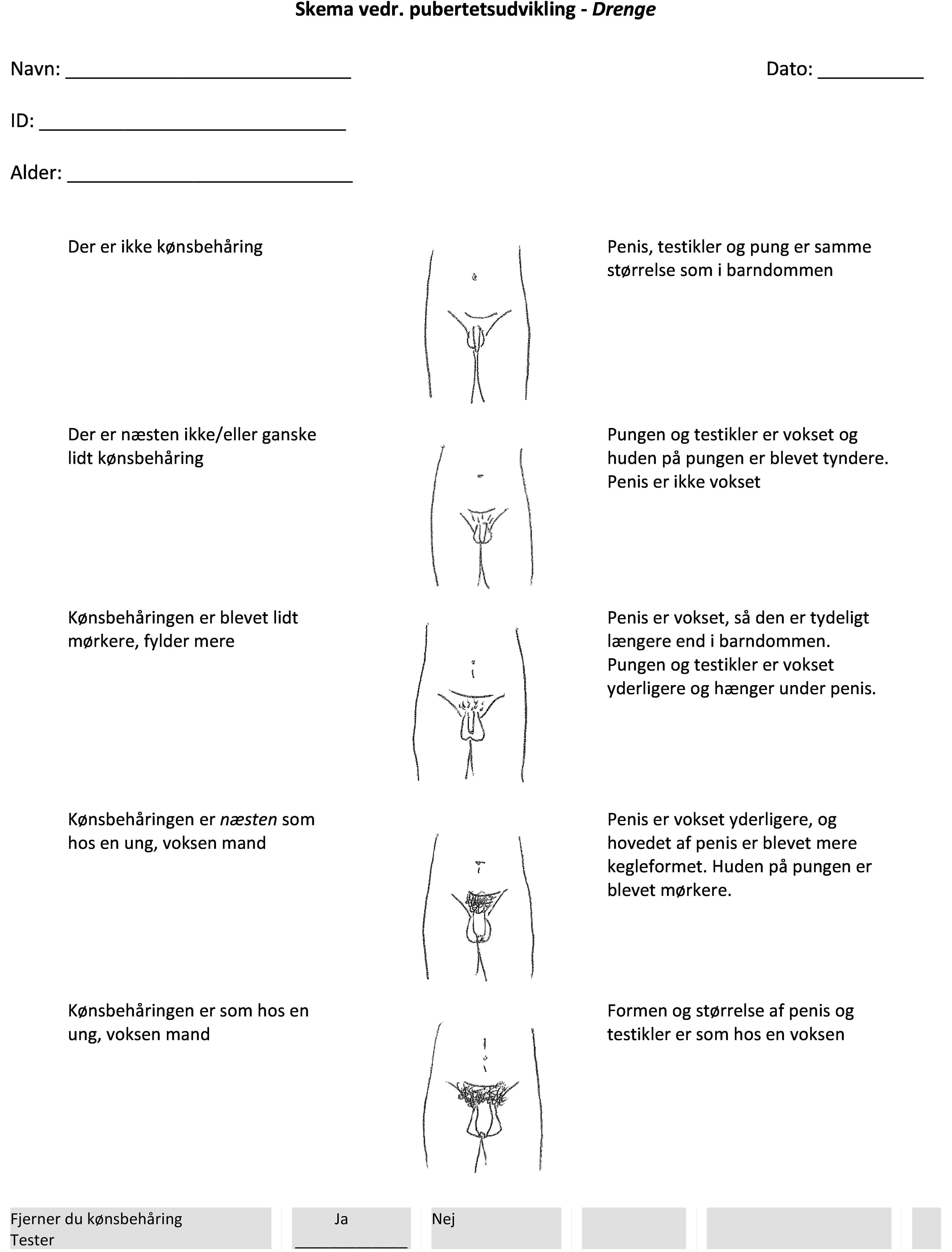

Supplement: S2 Appendix — (TIF) [file pone.0157182.s002.tif]
